# Azithromycin versus standard care in patients with mild-to-moderate COVID-19 (ATOMIC2): an open-label, randomised trial

**DOI:** 10.1016/S2213-2600(21)00263-0

**Published:** 2021-10

**Authors:** Timothy S C Hinks, Lucy Cureton, Ruth Knight, Ariel Wang, Jennifer L Cane, Vicki S Barber, Joanna Black, Susan J Dutton, James Melhorn, Maisha Jabeen, Phil Moss, Rajendar Garlapati, Tanya Baron, Graham Johnson, Fleur Cantle, David Clarke, Samer Elkhodair, Jonathan Underwood, Daniel Lasserson, Ian D Pavord, Sophie Morgan, Duncan Richards

**Affiliations:** a Respiratory Medicine Unit and National Institute for Health Research Oxford Biomedical Research Centre, Nuffield Department of Medicine Experimental Medicine, University of Oxford, Oxford, UK; b Oxford Clinical Trials Research Unit, Centre for Statistics in Medicine, Nuffield Department of Orthopaedics, Rheumatology and Musculoskeletal Sciences, Botnar Research Centre, University of Oxford, Oxford, UK; c University Hospital Llandough, Cardiff, UK; d Emergency Department Clinical Research Unit, St George's Hospital, London, UK; e Accident and Emergency, East Lancashire NHS Hospitals, Blackburn, UK; f Emergency Department, Oxford University Hospitals NHS Foundation Trust, Oxford, UK; g University Hospitals of Derby and Burton, Royal Derby Hospital, Derby, UK; h University of Nottingham, Lenton, Nottingham UK; i Department of Emergency Medicine, King's College Hospital, London, UK; j Royal Berkshire Hospital, Reading, UK; k Emergency Department, University College London Hospital, London UK; l Department of Infectious Diseases, Cardiff and Vale University Health Board, Cardiff, UK; m Division of Infection and Immunity, Cardiff University, Cardiff, UK; n Department of Geratology, Oxford University Hospitals NHS Foundation Trust, John Radcliffe Hospital, Oxford, UK; o Department of Acute Medicine, Sandwell and West Birmingham Hospitals NHS Trust, City Hospital, Birmingham, UK; p Warwick Medical School, University of Warwick, Coventry, UK

## Abstract

**Background:**

The antibacterial, anti-inflammatory, and antiviral properties of azithromycin suggest therapeutic potential against COVID-19. Randomised data in mild-to-moderate disease are not available. We assessed whether azithromycin is effective in reducing hospital admission in patients with mild-to-moderate COVID-19.

**Methods:**

This prospective, open-label, randomised superiority trial was done at 19 hospitals in the UK. We enrolled adults aged at least 18 years presenting to hospitals with clinically diagnosed, highly probable or confirmed COVID-19 infection, with fewer than 14 days of symptoms, who were considered suitable for initial ambulatory management. Patients were randomly assigned (1:1) to azithromycin (500 mg once daily orally for 14 days) plus standard care or to standard care alone. The primary outcome was death or hospital admission from any cause over the 28 days from randomisation. The primary and safety outcomes were assessed according to the intention-to-treat principle. This trial is registered at ClinicalTrials.gov (NCT04381962) and recruitment is closed.

**Findings:**

298 participants were enrolled from June 3, 2020, to Jan 29, 2021. Three participants withdrew consent and requested removal of all data, and three further participants withdrew consent after randomisation, thus, the primary outcome was assessed in 292 participants (145 in the azithromycin group and 147 in the standard care group). The mean age of the participants was 45·9 years (SD 14·9). 15 (10%) participants in the azithromycin group and 17 (12%) in the standard care group were admitted to hospital or died during the study (adjusted OR 0·91 [95% CI 0·43–1·92], p=0·80). No serious adverse events were reported.

**Interpretation:**

In patients with mild-to-moderate COVID-19 managed without hospital admission, adding azithromycin to standard care treatment did not reduce the risk of subsequent hospital admission or death. Our findings do not support the use of azithromycin in patients with mild-to-moderate COVID-19.

**Funding:**

National Institute for Health Research Oxford Biomedical Research Centre, University of Oxford and Pfizer.

## Introduction

Azithromycin is an orally active synthetic macrolide antibiotic with a wide range of antibacterial, anti-inflammatory, and antiviral properties.^1^ Early in 2020 it was highlighted by in silico and in vitro^2^ screens as a potential candidate therapy to be repurposed for treatment of COVID-19. Macrolides, particularly azithromycin, have previously been used to treat other viral infections, including one in three severe cases of MERS-CoV,^3^ although randomised controlled trial data for its use in any coronavirus disease were absent.^4^ Azithromycin is inexpensive, safe, and widely available, and—stimulated by a small, non-randomised clinical report^5^—its use in the context of COVID-19 has become widespread in clinical practice and clinical trials.

In vitro azithromycin has broad antiviral activity against human viruses, including human rhinovirus, Zika virus, enteroviruses, Ebola virus, SARS-CoV,^1^ and against SARS-CoV-2, being shown to reduce viral replication alone^2^ or in combination with hydroxychloroquine.^6^ Azithromycin was also associated with reduced viral load of non-SARS-CoV-2 alphacoronaviruses and betacoronaviruses in children receiving azithromycin during a mass distribution programme.^7^ Although antivirals probably have little efficacy in severe disease after viraemia has peaked,^8^, ^9^, ^10^ azithromycin does have anti-inflammatory properties, including dose-dependent suppression of lymphocyte expression of perforin, and a range of proinflammatory cytokines, including IL-1β, IL-6, and TNF, IL-8 (CXCL8), IL-18, G-CSF, and GM-CSF^11^, ^12^ and other components of the IL-1β–IL-6-induced acute phase response such as serum amyloid protein A.^12^

Despite these theoretical considerations, large-scale clinical trials of azithromycin either alone or co-administered with hydroxychloroquine have not shown clinical efficacy in reducing mortality, need for invasive mechanical ventilation, duration of hospital admission, or clinical status on ordinal outcome scores in patients admitted to hospital with COVID-19.^13^, ^14^, ^15^, ^16^ However, these trials were all done in late-stage, severe disease with 17–40% mortality. They did not study patients at earlier stages of disease in the community and are not able to make conclusions about the effectiveness of azithromycin outside the hospital setting. The efficacy of therapies in COVID-19 depends on the timing in the course of disease and in the populations being studied. Dexamethasone showed a strong survival benefit with treatment in patients with severe COVID-19 but no benefit, or even potential harm, in those not requiring oxygen therapy.^17^ Conversely, studies of neutralising antibodies showed benefits in early disease^18^ but not in patients admitted to hospital.^19^ The antiviral and anti-inflammatory properties of azithromycin are suited to earlier-stage disease; thus, we studied an ambulatory population to establish whether it averts disease progression.

**Research in context**

**Evidence before this study**

We searched MEDLINE and the Cochrane Central register of Controlled Trials with the terms (“azithromycin”) AND (“COVID” OR “COVID-19”) AND (“clinical trials”), until March 25, 2021, with no language restrictions. We identified 42 studies, among which there were four completed randomised trials of azithromycin (with or without hydroxychloroquine) in patients admitted to hospital with severe COVID-19 disease, and three completed randomised trials of azithromycin in patients with mild COVID-19 in primary care. The four randomised trials in patients admitted to hospital assigned 8988 participants to azithromycin or standard care or hydroxychloroquine and found no evidence of a difference in mortality, duration of hospital stay, or peak disease severity. The three trials in primary care settings randomly assigned participants with early disease to 3 days or 5 days of therapy, and only one assessed azithromycin as standalone therapy. That trial was a large, adaptive platform trial in the UK that randomly assigned 540 participants in primary care to 3 days of treatment with azithromycin and 875 participants to standard care alone and found no meaningful difference in time to first reported recovery or in rates of hospital admission (3% in both groups), and there were no deaths. We did not identify any randomised trials in patients with COVID-19 managed in ambulatory care.

**Added value of this study**

The ATOMIC2 trial was uniquely designed to assess azithromycin as a standalone therapy in those with mild-to-moderate COVID-19 presenting to emergency care but assessed as appropriate for initial ambulatory management without hospital admission. ATOMIC2 also uniquely assessed high-dose, long-duration treatment to investigate the efficacy of putative anti-inflammatory effects. We found that azithromycin 500 mg daily for 14 days did not reduce the proportion of participants who died or required hospital admission from any cause over the 28 days from randomisation compared with standard care.

**Implications of all the available evidence**

Our findings, taken together with existing data, suggest there is no evidence that azithromycin reduces hospital admission, respiratory failure, or death compared with standard care, either in early disease in the community, or those admitted to hospital with severe disease, or in those with moderate disease managed on an ambulatory pathway.

We did a randomised, open-label clinical trial to establish whether azithromycin is effective in preventing hospital admission or death in adult patients with clinically diagnosed COVID-19 infection being managed on an ambulatory care pathway.

## Methods

### Study design

ATOMIC2 was a prospective, open-label, two-arm, randomised superiority trial of standard care and azithromycin compared with standard care alone, done at 19 hospitals in the UK. The trial was done according to the published protocol, version 7.0 (appendix p 87)^20^ and all applicable laws and regulations including, but not limited to, the principles as stated in the International Council for Harmonisation Guideline for Good Clinical Practice, the standards set out by the Research Governance Framework, the Medicines for Human Use (Clinical Trials) Regulations 2004, and the ethical principles that have their origin in the Declaration of Helsinki. Safety data were reviewed and monitored by an independent data safety monitoring committee. The trial protocol was reviewed and approved by the UK Medicines and Healthcare products Regulatory Agency and an independent ethical committee (London—Brent Research Ethics Committee, reference number 20/HRA/2105).

### Participants

Eligible participants were adults aged at least 18 years assessed in an acute hospital with a clinical diagnosis of highly probable or confirmed COVID-19 infection made by the attending clinical team, with onset of first symptoms within the past 14 days, and assessed by the attending clinical team as appropriate for initial ambulatory (ie, outpatient) management. Attendance for assessment in secondary care will have occurred via referral from primary care, or a national helpline, or self-referral. Key exclusion criteria were known hypersensitivity to any macrolide or the excipients, fructose intolerance, glucose-galactose malabsorption or sucrose-isomaltase insufficiency, current therapy with a macrolide antibiotic, hydroxychloroquine, or chloroquine, significant myocarditis, prolongation of a corrected QT interval (QTc) of more than 480 msec, significant electrolyte disturbance, clinically relevant bradycardia, ventricular tachycardia, unstable severe cardiac insufficiency, and inability to understand written English. The full protocol including a complete list of inclusion and exclusion criteria are in the appendix (pp 112–13).^20^ All patients provided electronic informed consent before randomisation.

### Randomisation and masking

Patients were randomly assigned (1:1) to either azithromycin plus standard care or standard care alone using a web-based automated service, with a minimisation algorithm to ensure balanced allocation across treatment groups, stratified by centre, sex, and presence of hypertension and diabetes. To ensure the unpredictability of treatment allocation, the first 30 participants were randomly assigned by simple randomisation and the minimisation algorithm included a probabilistic element (participants had an 80% chance of being allocated to the treatment, which minimised imbalance between the groups). Patients, investigators, and health-care providers were not masked to study drug assignment.

### Procedures

Data on demographics, medical history, symptoms, risk factors for disease progression, and disease severity (age, male sex, hypertension, cardiovascular disease, diabetes, chronic lung disease, and cancer) were collected on all patients at baseline. Results were recorded for vital signs (temperature, respiratory rate, heart rate, blood pressure, and oxygen saturation), physical examination (including chest auscultation) with clinical assessment including the COVID Core Outcomes Set^21^ (a widely-used consensus set of clinical outcomes arising from a Delphi survey), and a nine-level severity score of respiratory illness (0–8, where 0 indicates “Ambulatory. No limitation of activities” and 8 indicates “Death”). Participants also had a 12-lead electrocardiogram. Optional study samples were taken at baseline and on one further occasion if the participant was admitted: oropharyngeal swab for SARS-CoV-2 PCR and nasal and blood samples for RNA transcriptomic analysis. Bloods and chest x-rays were done if clinically required.

Patients in the azithromycin group received 500 mg azithromycin once daily orally plus standard care for 14 days and those in the control group received standard care according to local guidelines. Use of corticosteroids, other immunomodulators, antibiotics, and antivirals was permitted after randomisation, but the protocol excluded concomitant use of quinolone or macrolides antibiotics at enrolment or during follow-up. Subsequent assessments were conducted by telephone at days 14 and 28, and radiology results and clinical notes were assessed daily during hospital admission if this occurred.

### Outcomes

The primary outcome was the proportion of participants with hospital admission or death from any cause within 28 days from randomisation.

Secondary outcomes were the proportion of participants with hospital admission with respiratory failure requiring non-invasive mechanical ventilation or invasive mechanical ventilation or death from any cause within 28 days from randomisation; the proportion of participants with hospital admission with respiratory failure requiring invasive or non-invasive mechanical ventilation support or death from any cause over 28 days from randomisation among those with a PCR-confirmed diagnosis of COVID-19 at randomisation; mortality or all-cause hospital admission amongst those with a PCR-confirmed diagnosis; all-cause mortality at day 28; the proportion progressing to clinician-diagnosed pneumonia; the proportion progressing to severe pneumonia; and differences in the peak severity of respiratory illness according to a nine-level ordinal severity score for clinical condition (appendix p 116). We also assessed safety and tolerability of azithromycin based on serious adverse events.

### Statistical analysis and protocol changes

We had originally planned for the trial to recruit up to 800 participants and use the following primary outcome: the proportion of participants with hospital admission with respiratory failure requiring non-invasive mechanical ventilation or invasive mechanical ventilation or death from any cause within 28 days from randomisation. However, a decision was made at the preplanned interim analysis of 109 participants reaching the 28-day post-randomisation timepoint to update the primary outcome, because no primary outcome events had occurred at that stage. This implied that the original primary outcome, specified early in the pandemic when data on hospital admission rates in this population were unknown, proved incorrect for this population. The change was adopted in line with advice from the data safety monitoring committee and in accordance with the recommendations of the WHO Blueprint for COVID-19 Therapeutic Trials^22^ that the primary endpoint should be responsive to the eligible patient population and the definition of the endpoint should be fine-tuned for the pivotal phase, based on the pilot phase of the trial. This change was approved by the research ethics committee and the Medicines and Healthcare products Regulatory Agency on Feb 4, 2021, and implemented before final analyses were done. Two other protocol amendments were made while the trial was ongoing to broaden inclusion criteria to include and ensure the safety of participants taking selective serotonin reuptake inhibitors (appendix p 144).

Based on WHO recommendations that a pilot phase with 100 patients would be sufficient to inform follow-on clinical research,^22^ an interim analysis was planned to establish a definitive sample size. Following the revision to the primary outcome and based on masked data from the pilot phase, the definitive sample size was determined. Assuming a 15% rate of all-cause hospital admission or death in the standard care group, we estimated that a minimum of 276 participants providing primary endpoint data would provide 80% power and 5% (two-sided) significance to detect a difference from 15% to 5% in the azithromycin group, a relative reduction of 66%. To allow for 5% loss to follow-up we therefore aimed to recruit a minimum of 291 participants. The full statistical analysis plan is in the appendix (pp 40–86).

For the primary outcome, the difference in proportions between the treatment groups was assessed using a χ^2^ test and a 5% (two-sided) significance level. Adjusted analysis was done using logistic regression with progression as the binary outcome, adjusting for the following stratification factors: centre, hypertension, diabetes, and sex. A supporting analysis was also done to further adjust for the following important prognostic variables: age 65 years and older, presence of chronic lung disease, and treatment for cancer. Time-to-event analysis was also done to explore whether the active treatment delays progression. The success of the trial was based on the adjusted analysis. Both relative and absolute differences in proportions are reported together with 95% CIs. Other binary outcomes were assessed using similar methods. Peak severity of illness was considered a categorical variable and assessed using ordinal logistic regression analysis. The change in severity scale score from baseline was summarised on a continuous scale using means, SDs, medians, IQRs, and ranges. We explored consistency of results for the following prespecified stratification factors: hypertension, diabetes, sex, and age using treatment by variable interaction tests and forest plots. Analyses were done using Stata IC, version 15.1.

Primary and safety analyses were based on the intention-to-treat (ITT) population, defined as all randomly assigned patients analysed according to their randomised allocation. A supplementary ITT population (ITT positive) was defined as all randomly assigned patients with a positive baseline COVID-19 test based on baseline swabs. This trial was registered with ClinicalTrials.gov (NCT04381962) and EudraCT (2020-001740-26).

### Role of the funding source

The funder of the study had no role in study design, data collection, data analysis, data interpretation, or writing of the report.

## Results

**Figure 1:**
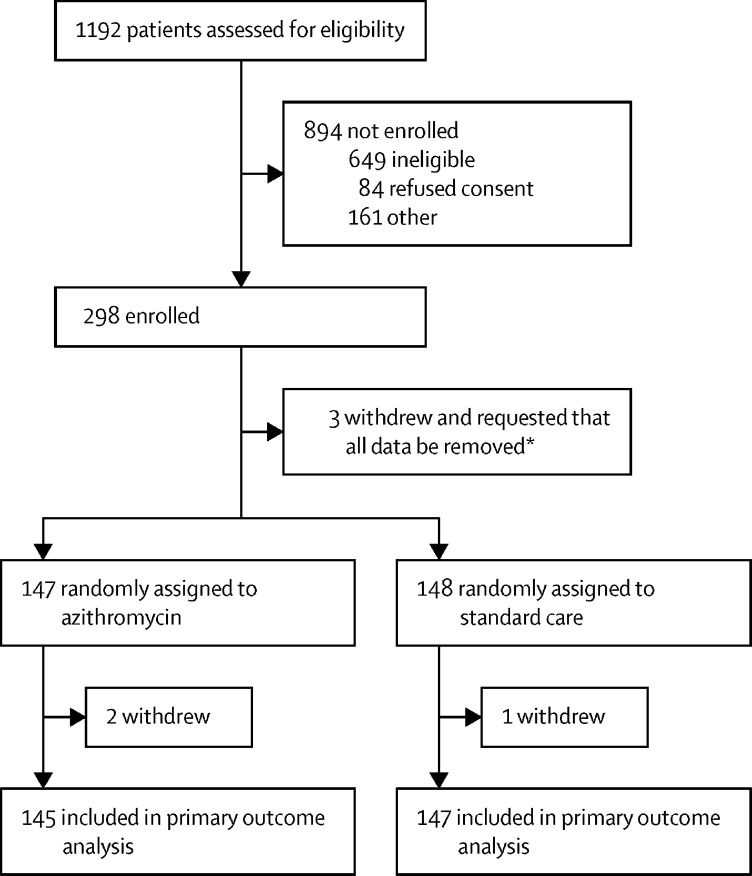
Trial profile *These participants withdrew completely and asked for all their data collected to date to be removed; therefore, they have not been included in any summaries or analyses.

From June 3, 2020, to Jan 29, 2021, 1192 patients were screened, of whom 298 were enrolled in the trial (figure 1; appendix p 10). Three participants withdrew consent and requested removal of all data collected so are not presented in baseline data. Of the 295 participants, 147 were randomly assigned to azithromycin plus standard care and 148 were randomly assigned to standard care alone. Three of 295 patients withdrew consent after randomisation (appendix p 23); thus, data on the primary outcome were available from 292 participants. Deviations from protocol are shown in the appendix (p 24).

Characteristics of participants assigned to azithromycin and to standard care were similar (table 1; appendix pp 12–22). The mean participant age was 45·9 years (SD 14·9); 152 (52%) of 295 were men and 143 (49%) were women; 201 (68%) were White, 47 (16%) were Asian or Asian British, 11 (4%) were Black or Black British, and 36 (12%) were Mixed or other race; 70 (24%) participants had comorbidities; and the median duration of symptoms before enrolment was 6·02 days (3·52). 246 (83%) participants had cough and 225 (76%) had dyspnoea at presentation (appendix p 18). Enrolment was based on a clinical diagnosis of highly probable COVID-19, but the definitive results of nasopharyngeal swabs for SARS-CoV-2 PCR were available from 231 individuals, of which 152 (66%) were positive, and constituted the ITT positive population (76 azithromycin and 76 standard care). 143 (97%) of 147 participants allocated to azithromycin commenced treatment, with 76 (52%) achieving full compliance (taking a median 28 tablets [IQR 28–28]), 51 (35%) non-compliant, taking a median 6 tablets (IQR 2–17), and compliance was unknown in 20 (14%; appendix p 23).

**Table 1:** Baseline characteristics and concomitant treatments of the intention-to-treat population

		**Azithromycin group (n=147)**	**Standard care group (n=148)**
Hypertension			
	Yes	25 (17%)	27 (18%)
	No	122 (83%)	121 (82%)
Diabetes			
	Yes	11 (7%)	14 (9%)
	No	136 (93%)	134 (91%)
Gender			
	Men	76 (52%)	76 (51%)
	Women	71 (48%)	72 (49%)
Age, years		45·5 (14·2)	46·3 (15·5)
COS*		6·36 (3·64)	7·00 (3·87)
COS Plus*		7·66 (4·65)	8·86 (5·25)
Duration of symptoms, days		5·77 (3·49)	6·27 (3·55)
Ethnicity			
	White	103 (70%)	98 (66%)
	Asian or Asian British	23 (16%)	24 (16%)
	Black or Black British	6 (4%)	5 (3%)
	Mixed	0	4 (3%)
	Other†	15 (10%)	17 (11%)
Smoking			
	Never smoked	81 (55%)	76 (51%)
	Former smoker	25 (17%)	26 (18%)
	Current smoker	16 (11%)	17 (11%)
	Former smoker and current vaper	3 (2%)	4 (3%)
	Never smoked and current vaper	0	1 (1%)
	Not recorded	16 (11%)	19 (13%)
Residence			
	Non-residential care	132 (90%)	137 (93%)
	Residential care	7 (5%)	3 (2%)
	No fixed address	5 (3%)	4 (3%)
Live alone			
	Yes	17 (12%)	13 (9%)
	No	108 (73%)	110 (74%)
Work status			
	Retired	15 (10%)	23 (16%)
	Working	101 (69%)	95 (64%)
	Not working	22 (15%)	21 (14%)
Occupation			
	Not health-care related	77 (52%)	69 (47%)
	Health-care worker	20 (14%)	23 (16%)
	Laboratory worker	1 (1%)	1 (1%)
Comorbidities			
	None	107 (73%)	107 (72%)
	At least one	33 (22%)	37 (25%)
Asthma			
	Yes	26 (18%)	27 (18%)
	No	121 (82%)	121 (82%)
History of previous myocardial infarction			
	Yes	5 (3%)	7 (5%)
	No	142 (97%)	141 (95%)
Currently undergoing any cancer treatment			
	Yes	1 (1%)	0
	No	146 (99%)	148 (100%)
Chronic pulmonary disease			
	Yes	7 (5%)	5 (3%)
	No	140 (95%)	143 (97%)
Severity scale score‡			
	Ambulatory, no limitation of activities	61 (41%)	66 (45%)
	Limitation of simple activities	85 (58%)	81 (55%)
	Admitted to hospital, mild disease, no oxygen therapy	1 (1%)	1 (1%)
Pneumonia§			
	Yes	28 (19%)	34 (23%)
	No	119 (81%)	114 (77%)
Swab results¶			
	Positive	76 (52%)	76 (51%)
	Negative	41 (28%)	38 (26%)
	Failed assay	0	3 (2%)
	Not available	30 (20%)	31 (21%)

Data are n (%) or mean (SD). COS=core outcome set.

* COVID-19 COS score of clinical symptoms is a total score of six common and important clinical symptoms, each scored as 0 (no), 1 (mild), 2 (moderate), or 3 (significant); an amended version, COS PLUS, contains an additional two clinical symptoms, anosmia and dysgeusia; COS scores range 0–18 and COS Plus scores 0–24, with higher scores indicating more severe symptoms.

† Other includes Chinese, any other ethnic group, and not stated.

‡ Severity scale scores range 0–8, with higher scores indicating the most severe status, death.

§ Pneumonia is defined as consolidation on a chest x-ray, if a chest x-ray was not taken it is assumed there was no pneumonia.

¶ Swab test results are only available for those who had a COVID-19 swab at randomisation.

**Figure 2:**
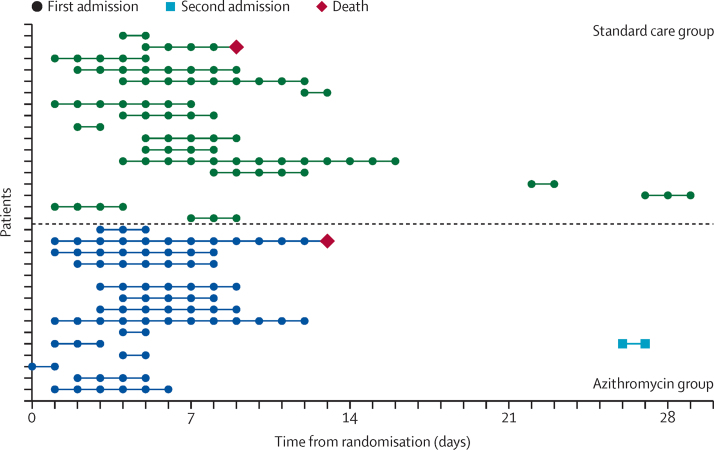
Time to hospital admission, length of stay, and time to death in the 32 participants admitted to hospital during the study One participant in the azithromycin group was known to have been admitted to hospital but exact dates were not available so details of their stay could not be included in the plot.

**Figure 3:**
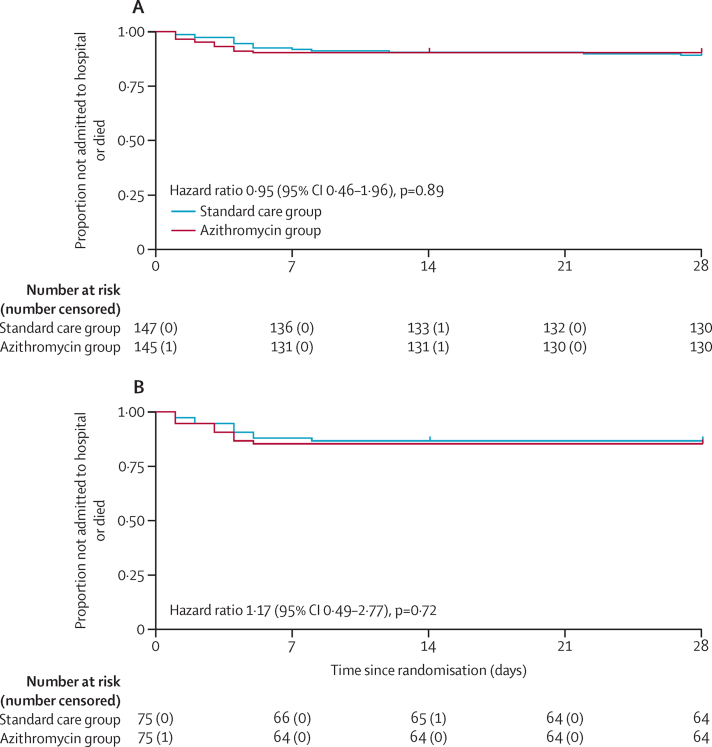
Kaplan-Meier plot of time to hospital admission in the intention-to-treat population (A) and in the intention-to-treat positive population (B)

15 (10%) of 145 participants randomly assigned to azithromycin and 17 (12%) of 147 participants randomly assigned to standard care were admitted to hospital or died. The primary endpoint, using the prespecified adjusted analysis, was not significantly different between the azithromycin and control groups (adjusted OR 0·91 [95% CI 0·43–1·92], p=0·80; table 2; appendix pp 24–25). There was no difference in the time to hospitalisation (adjusted hazard ratio [HR] 0·95 [95% CI 0·46–1·96], p=0·89; table 2, Figure 2, Figure 3; appendix p 26). In the ITT positive population there were also no differences in the combined primary outcome of hospitalisation or death (adjusted OR 1·02 [0·40–2·57], p=0·97) or time to hospitalisation (HR 1·17 [0·49–2·77]), p=0·72; figure 3B, table 2). Additionally, unadjusted and fully adjusted analyses (further adjusted for age, chronic pulmonary disease, and presence of cancer) were done, as well as analyses in the per-protocol population. None of these analyses demonstrated significant differences between the treatment groups (appendix pp 37–38). We did not observe heterogeneity of effect across subgroups with risk factors for severe disease (older age, male sex, hypertension, and diabetes; appendix p 38).

**Table 2:** Comparison of primary and secondary binary outcomes, and time-to-event in the ITT populations

		**Azithromycin group**	**Standard care group**	**Comparison of proportions**		**Time-to-event, hazard ratio (95% CI), p value**
				Odds ratio (95% CI), p value	Risk difference (95% CI)	
**Primary outcome**						
Hospitalisation or death (ITT)		15/145 (10%)	17/147 (12%)	..	..	..
	Unadjusted	..	..	0·88 (0·42 to 1·84), 0·74	−1·2% (−8·4 to 5·9)	0·79
	Adjusted	..	..	0·91 (0·43 to 1·92), 0·80	−1·0% (−8·0 to 6·1)	0·95 (0·46 to 1·96), 0·89
	Fully adjusted	..	..	0·91 (0·42 to 1·97), 0·82	−1·2% (−8·2 to 5·7)	0·99 (0·49 to 2·00), 0·99
Hospitalisation or death (ITT positive)		11/75 (15%)	11/75 (15%)	..	..	..
	Unadjusted	..	..	1·00 (0·40 to 2·47), 1·00	0·0% (−11·3 to 11·3)	0·78
	Adjusted	..	..	1·02 (0·40 to 2·57), 0·97	0·3% (−10·8 to 11·4)	1·17 (0·49 to 2·77), 0·72
	Full adjusted	..	..	1·11 (0·43 to 2·90), 0·83	0·8% (−10·1 to 11·7)	1·30 (0·52 to 3·21), 0·57
**Secondary outcomes**						
Level 2 or 3 ventilation or death		2/145 (1%)	2/147 (1%)	p=1·00	..	..
All-cause mortality		1/145 (1%)	1/147 (1%)	p=1·00	..	..
Progression to pneumonia		0/119	2/114 (2%)	p=0·24	..	..

Comparisons for the primary outcome were performed using a logistic regression model adjusted for stratification factors centre, hypertension, diabetes, and sex (adjusted), or fully adjusted for centre, hypertension, diabetes, sex, age 65 years and older, presence of chronic lung disease, and treatment for cancer. Comparisons for the secondary outcomes were performed using Fisher's exact test because of small numbers of events. ITT=intention to treat.

Two participants (1%) in each group required level 2 or 3 ventilation or died. Because of the very low number of events, Fisher's exact test was used to compare the azithromycin and control groups, and results showed no significant differences (table 2). The level of oxygen support required by each participant during hospital admission is summarised in the appendix (p 27). The number of deaths from all-causes and participants progressing to pneumonia were similarly very low with no differences between the two groups (table 2). No participants progressed to severe pneumonia during the trial. Full details of pneumonia status at baseline and follow-up are in the appendix (p 28). Analyses of these outcomes were also repeated on the ITT positive population, with no significant differences (appendix p 26).

**Figure 4:**
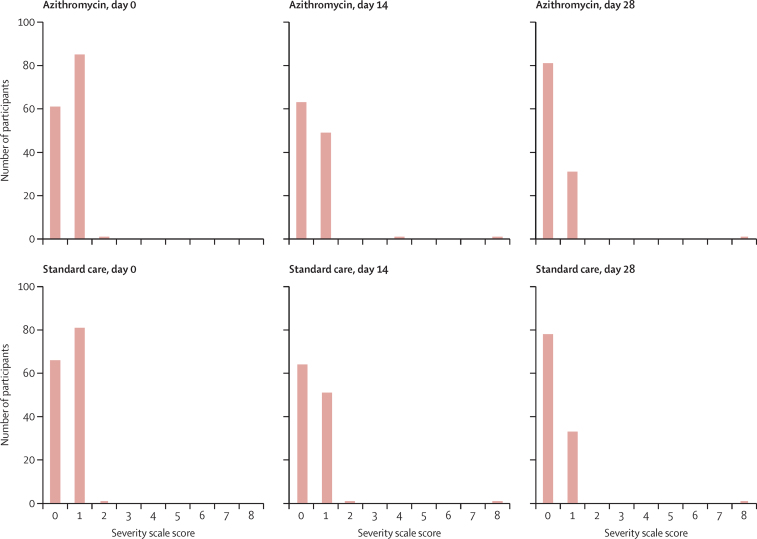
Severity scores at days 0, 14, and 28 in 254 participants with complete data from the intention-to-treat population

Most participants had severity scores of 0 or 1 at baseline and at days 14 and 28 (figure 4; appendix pp 29–30). The peak severity score during follow-up was calculated for each participant with data available. 62 (50%) of 124 participants randomly assigned to azithromycin and 60 (46%) of 130 participants randomly assigned to standard care reported no limitation of activities as their highest follow-up severity score (table 3; appendix pp 31–33). 49 (40%) in the azithromycin group and 57 (44%) in the standard care group reported limitation of simple activities as their peak score. There was no significant difference between the two groups in terms of peak severity score (adjusted OR 0·91 [95% CI 0·57–1·46], p=0·69). The analysis was repeated for the ITT positive population, with no significant differences observed (appendix p 32). In an exploratory analysis, the COVID-19 Core Outcome Set Plus scores were similar in the two groups at 14 days (mean score 7·62 [SD 5·11] in the azithromycin group *vs* 7·47 [5·19] in the standard care group; ITT population) and 28 days (3·06 [3·61] *vs* 3·38 [3·52]; ITT population) after randomisation (appendix pp 34–36).

**Table 3:** Comparison of peak severity scores in the intention-to-treat-population

	**Azithromycin group (n=124)**	**Standard care group group (n=130)**
Ambulatory, no limitation of activities	62 (50%)	60 (46%)
Limitation of simple activities	49 (40%)	57 (44%)
Admitted to hospital, mild disease, no oxygen therapy	3 (2%)	2 (2%)
Admitted to hospital, oxygen by conventional delivery system ≤40% mask or nasal prongs	5 (4%)	10 (8%)
Admitted to hospital, oxygen by conventional delivery system >40% mask	3 (2%)	0
Admitted to hospital, receiving non-invasive mechanical ventilation or receiving high-flow oxygen therapy (>15 L/min), or continuous positive airway pressure	1 (1%)	1 (1%)
Death	1 (1%)	1 (1%)

Data are n (%). The adjusted* odds ratio for peak severity scores was 0·91 (95% CI 0·57–1·46), p=0·69.

* Adjusted for the stratification factors centre, hypertension, diabetes, and sex.

During follow-up, additional antibiotics were co-prescribed in 23 (16%) of 147 participants in the azithromycin group and 38 (26%) of 148 in the standard care group (appendix p 39). Use of inhaled or systemic corticosteroids was not recommended in contemporary UK guidelines for those not admitted to hospital, although inhaled corticosteroids were co-prescribed in nine (6%) participants receiving azithromycin and 19 (13%) receiving standard care, while systemic corticosteroids were co-prescribed in 16 (11%) receiving azithromycin and 16 (11%) receiving standard care (appendix p 39). No serious adverse events were recorded in either treatment group during follow-up. Three (2%) of 145 participants randomly assigned to azithromycin and four (3%) of 147 participants randomly assigned to standard care reported a complication during hospital stay (appendix p 37).

## Discussion

In this trial of people with clinically diagnosed mild-to-moderate COVID-19 managed without hospital admission, adding azithromycin to standard care treatment did not reduce the risk of subsequent hospital admission or death, or of time to hospital admission.

Three recent, large, open-label, randomised controlled trials have assessed the use of azithromycin in patients admitted to hospital with severe COVID-19, and none have found a clinically significant benefit in those populations. COALITION I randomly assigned 667 patients admitted to hospital with COVID-19 to standard care, hydroxychloroquine, or hydroxychloroquine with azithromycin 500 mg for 7 days and found no difference in clinical status on an ordinal score at 15 days between hydroxychloroquine with azithromycin and hydroxychloroquine (OR 0·82 [95% CI 0·47–1·43]; p=1·00).^16^ Likewise, COALITION II randomly assigned 447 patients admitted to hospital to azithromycin 500 mg daily for 10 days or standard care, and again found no difference in day 15 ordinal score (OR 1·36 [0·94–1·97], p=0·11).^13^ RECOVERY randomly assigned 7763 of its participants to azithromycin 500 mg for 10 days or standard care and found no difference in 28-day mortality (rate ratio 0·97 [95% CI 0·87–1·07], p=0·50), length of stay, or invasive mechanical ventilation and death.^15^ However, none of these trials assessed the potential for efficacy in early, milder disease.

Three trials in primary care have randomly assigned participants with early disease to 3 days^23^ or 5 days^24^, ^25^ of therapy. Azithromycin was assessed as standalone therapy only in the PRINCIPLE trial;^23^ a large, adaptive platform trial in the UK, which randomly assigned 540 participants to 3 days treatment with azithromycin 500 mg daily versus 875 participants to standard care. This study found no difference in time to first reported recovery (HR 1·08 [95% Bayesian credibility interval [BCI] 0·95 to 1·23]), and, although only 3% of participants were admitted to hospital, there was no significant difference between groups (absolute benefit in 0·3% [95% BCI −1·7 to 2·2]). The remaining two trials also used short courses of azithromycin 500 mg for 1 day followed by 250 mg for 4 days and taken in conjunction with hydroxychloroquine. Q-PROTECT recruited healthy, SARS-CoV-2-positive men in a quarantine site in Qatar and found no difference in time to virological cure (p=0·82),^24^ with low rates of hospital admission in all groups (2·4%). A study in the USA assessed progression to lower respiratory tract infection, hospital admission, or death and time to viral clearance in SARS-CoV-2-positive outpatients, but was stopped early for futility because of a low rate of clinical outcomes in this population, and found no difference in the co-primary outcome of time to virological clearance (HR 1·25 [95% CI 0·75 to 2·07], p=0·39).^25^ No studies have assessed azithromycin in patients presenting to hospital with substantial symptoms, but early enough in the disease process to be managed in ambulant care, and neither have previous studies assessed high-dose, long-duration azithromycin therapy in early disease.

Our study investigated this intermediate population with early disease, but at high risk of deterioration, in whom 11% required subsequent hospital admission. Therefore, our population represents those with the optimal chance of demonstrating clinical benefit in early disease. We did not observe a significant difference in our primary outcome. Given the small absolute event rates for the primary outcome in our study, a smaller but clinically relevant effect cannot be entirely ruled out, but would be unlikely to change clinical practice. Nonetheless, this finding, taken together with clear negative results across the disease course from early, low-risk patients, to patients admitted to hospital with severe disease, provides strong confirmation that azithromycin is not effective in treating COVID-19.

A unique feature of ATOMIC2 was its successful implementation at the interface between community and secondary care, which is often a challenging location to recruit due to pressures for rapid clinical decision making. This implementation was made possible by electronic screening, consent, and recruitment. This platform, along with simplicity and broad inclusion criteria, have facilitated a second strength of the study: inclusion of an ethnically diverse population, with 32% of participants recruited from Black and minority ethnic (BAME) backgrounds. This is important as COVID-19 is a global pandemic, with a disproportionate impact on BAME groups, yet these groups are under-represented in many COVID-19 trials, which might reduce external validity.^26^ Another strength of our study is that in contrast to other studies, the high dose (500 mg daily) and long duration (14 days) of azithromycin was used to ensure that we adequately assessed potential antiviral, antibacterial, and anti-inflammatory benefits. COVID-19 is considered to have a distinct early viraemic phase and a late inflammatory phase in some individuals, and therefore assessment of antiviral activity needs to be early in the disease course before onset of severe disease.^20^ Conversely it was not known what doses might be required to produce an adequate anti-inflammatory effect so it was necessary to give a high dose of long duration to ensure the anti-inflammatory effect was tested throughout the late stage of innate or acute phase inflammatory cytokine dysregulation.^1^ An additional strength is that we were also able to exclude a significant benefit from azithromycin's antibiotic effects, which was not possible in studies of patients in hospital where co-prescribing of β-lactam and other antibiotics was common.^15^ Our data show that secondary bacterial infection is not a major driver of hospital admission in this population.

A limitation of our trial is that it was open-label, because of the difficulty obtaining appropriate placebos early in the pandemic and is therefore at risk of bias particularly on patient-reported outcomes. However, our choice of hospital admission and death as the primary outcome is unlikely to be markedly influenced by selection, detection or observer bias. Detection of admission to hospital has been easier because of restricted movement during lockdowns, from use of regional and national electronic health records, and by systematic contacting of all participants, with more than 95% follow-up. Moreover, a placebo effect from perceived benefits of treatment would tend towards a positive effect of azithromycin, which was not observed. Knowledge of treatment allocation might also lead to a tendency to more antibiotic prescribing in the placebo group—an effect we observed—which would tend to prejudice against azithromycin as an antibiotic, although concomitant use of macrolides specifically was prohibited by the protocol. A second limitation is that, like other studies,^23^ we used a clinical diagnosis for inclusion, rather than requiring PCR confirmation, and PCR data were not available for all participants—particularly at the early stages of the pandemic in the UK where low testing capacity was directed to patients who needed admission to hospital. While it is likely some participants who did not ultimately have COVID-19 might have been enrolled, this decision reflects the situation in many urgent care settings globally where PCR confirmation is not immediately available, and enhances the generalisability of our findings. Nonetheless, SARS-CoV-2 was detected in 66% of those with successful PCR assays in our study, which is much higher than the 31% PCR-positive rate observed in PRINCIPLE,^23^ and study results were similar in the overall ITT group and the predefined PCR-positive subgroup analysis. An ITT analysis was selected as the most appropriate approach to establish whether this intervention affects clinical outcomes. While throat swab PCR assays have high specificity, they have low sensitivity in routine clinical practice, and consequently where there is a high pre-test probability for COVID-19, as was the situation in those enrolled, a negative PCR result has a low negative predictive value and most negative results will be false negatives.^27^ A third limitation is the relatively young mean age of the study population (45·9 years), which reduces the proportion who are likely to have severe disease. Nonetheless, the primary outcome occurred in more than 10% of participants, and globally, individuals of similar age could have been receiving azithromycin therapy in many countries. Other limitations are incomplete compliance to the long treatment regimen in some individuals and absence of data on microbiology or long-term outcomes beyond 28 days.

In the past year, more than 40 clinical trials of azithromycin in COVID-19 have been registered.^1^ Given positive data from in silico and in vitro^2^ screens and data showing suppression of innate inflammatory cytokines in vitro, and clinical trial data in non-SARS-CoV-2 alphacoronaviruses and betacoronaviruses,^7^ why might these have not translated into clinical efficacy? Other antiviral molecules have had little clinical effect in COVID-19 compared with immunosuppressive therapies, except in very early disease. In common with influenza, antivirals are probably only efficacious in the early viraemic disease stage and are ineffective in severe disease, which is more closely linked to differences in host immune factors. In contrast to influenza A pandemics, the antibacterial effects of azithromycin are unlikely to translate into significant clinical benefit in a disease where secondary bacterial pneumonia is rare.^28^ While many studies have shown azithromycin suppression of innate cytokines known to be key mediators of severe disease, including IL-1β, IL-6, CXCL-8, TNF, and GM-CSF, some of these data might be confounded by antibacterial effects in the original studies, and it might be that the suppression achieved by azithromycin is simply insufficient to overcome the overwhelming cytokine production triggered by this virus in susceptible individuals.

We found no evidence of safety concerns associated with azithromycin, despite the quite high dose and long course prescribed. In particular, there were no adverse cardiac events; a concern raised by studies of azithromycin and hydroxychloroquine co-prescribing.^29^ In a Danish cohort analysis of 10·6 million prescriptions, azithromycin prescribing has been associated cardiovascular death (rate ratio 2·85 [95% CI 1·13–7·24]) compared with no antibiotics, but this is probably because of the underlying indication because, when compared with penicillin V, there was no increased risk once adjusted for propensity scores (rate ratio 0·93 [0·56–1·55]).^30^ This analysis was performed in a population with a low baseline risk of cardiovascular death, and it should be noted that we excluded patients with a prolonged QT interval at baseline electrocardiogram. Nonetheless, there are considerable population risks of unwarranted prescribing of azithromycin, which is a highly valuable antimicrobial and yet has a particularly high propensity for inducing antimicrobial resistance, both to macrolides and to other drug classes, including β-lactam antibiotics.^31^

In conclusion, our findings in mild-to-moderate COVID-19 managed in ambulatory care, taken together with trials in early disease in primary care and from trials in patients admitted to hospital with severe disease, suggest that azithromycin does not reduce hospital admissions, respiratory failure, or death compared with standard care, and should not be used in the treatment of COVID-19.

## Data sharing

The data analysed and presented in this study are available from the corresponding author on reasonable request, providing the request meets local ethical and research governance criteria after publication. Patient-level data will be anonymised and study documents will be redacted to protect the privacy of trial participants. The study protocol is in the appendix (pp 87–146).

## Declaration of interests

TSCH has received grants from Pfizer, University of Oxford, the Wellcome Trust, The Guardians of the Beit Fellowship, and the National Institute for Health Research (NIHR) Oxford Biomedical Research Centre (BRC) during the conduct of the study; and personal fees from Astra Zeneca, TEVA, and Peer Voice, outside of the submitted work. MJ has received grants from the University of Oxford and NIHR Oxford Biomedical Research Centre. DR has undertaken paid consultancy for GlaxoSmithKline outside of the submitted work. IDP reports personal fees from AstraZeneca, Boehringer Ingelheim, Aerocrine, Almirall, Novartis, GlaxoSmithKline, Genentech, Regeneron, Teva, Chiesi, Sanofi, Circassia, and Knopp; and grants from NIHR, outside of the submitted work. JU has received honoraria for preparation of educational materials and has served on an advisory board for Gilead Sciences and ViiV Healthcare, outside of the submitted work. All other authors declare no competing interests.
