# Correction to *Lancet Respir Med* 2021; 9: 1130–40

**DOI:** 10.1016/S2213-2600(21)00374-X

**Published:** 2021-10

*Hinks TSC, Cureton L, Knight R, et al. Azithromycin versus standard care in patients with mild-to-moderate COVID-19 (ATOMIC2): an open-label, randomised trial.* Lancet Respir Med *2021; **9:** 1130–40*—The appendix of this Article has been corrected and the data in the following Results text is now corrected: “…while systemic corticosteroids were co-prescribed in 13 (9%) receiving azithromycin”. These corrections have been made to the online version as of Sept 2, 2021, and will be made to the printed version.

